# SpliceMiner: a high-throughput database implementation of the NCBI Evidence Viewer for microarray splice variant analysis

**DOI:** 10.1186/1471-2105-8-75

**Published:** 2007-03-05

**Authors:** Ari B Kahn, Michael C Ryan, Hongfang Liu, Barry R Zeeberg, D Curtis Jamison, John N Weinstein

**Affiliations:** 1 Department of Bioinformatics, George Mason University, Fairfax, Virginia, USA; 2 Laboratory of Molecular Pharmacology, National Cancer Institute, National Institutes of Health, Bethesda, Maryland, USA; 3 Tiger Team Consulting, Fairfax, VA, USA; 4 Georgetown University, Washington, DC, USA; 5 School of Informatics, Northern Kentucky University, Highland Heights, KY, USA

## Abstract

**Background:**

There are many fewer genes in the human genome than there are expressed transcripts. Alternative splicing is the reason. Alternatively spliced transcripts are often specific to tissue type, developmental stage, environmental condition, or disease state. Accurate analysis of microarray expression data and design of new arrays for alternative splicing require assessment of probes at the sequence and exon levels.

**Description:**

SpliceMiner is a web interface for querying Evidence Viewer Database (EVDB). EVDB is a comprehensive, non-redundant compendium of splice variant data for human genes. We constructed EVDB as a queryable implementation of the NCBI Evidence Viewer (EV). EVDB is based on data obtained from NCBI Entrez Gene and EV. The automated EVDB build process uses only complete coding sequences, which may or may not include partial or complete 5' and 3' UTRs, and filters redundant splice variants. Unlike EV, which supports only one-at-a-time queries, SpliceMiner supports high-throughput batch queries and provides results in an easily parsable format. SpliceMiner maps probes to splice variants, effectively delineating the variants identified by a probe.

**Conclusion:**

EVDB can be queried by gene symbol, genomic coordinates, or probe sequence *via *a user-friendly web-based tool we call SpliceMiner (). The EVDB/SpliceMiner combination provides an interface with human splice variant information and, going beyond the very valuable NCBI Evidence Viewer, supports fluent, high-throughput analysis. Integration of EVDB information into microarray analysis and design pipelines has the potential to improve the analysis and bioinformatic interpretation of gene expression data, for both batch and interactive processing. For example, whenever a gene expression value is recognized as important or appears anomalous in a microarray experiment, the interactive mode of SpliceMiner can be used quickly and easily to check for possible splice variant issues.

## Background

There is a substantial difference between the number of genes in the human genome and the number of expressed transcripts and proteins. Alternative splicing largely accounts for that discrepancy. Based on experimental evidence and computational approaches (*e.g*. realignments of transcripts or hidden Markov models), the percentage of genes that exhibit alternative splicing has been estimated as anywhere from 30% to 99% [1,2]. Numerous reviews describe general aspects of alternative splicing [3-11], mechanisms of alternative splicing [12,13], and the roles played by alternative splicing in particular biological processes and diseases [14-24].

Until recently, microarray analysis has frequently assumed that transcript expression could be understood on the basis of gene-level information. However, splice variation is functionally important, and it can impact hybridization (*e.g*., to microarrays). A probe may, for example, target a sequence that is absent from a particular variant; that situation may lead to under-estimation of gene expression. Most existing traditional microarray platforms do not explicitly and systematically account for alternative splicing. Ideally, microarrays would include probes for each exon and splice site of each target gene to permit analysis of expressed splice forms.

Once a microarray has been manufactured, we cannot go back and change the design, but we can improve the analysis and interpretation of the results obtained from it. Furthermore, the annotation of newer microarrays designed to take alternative splicing into account will become inaccurate and obsolete as more information is deposited in the major genomic data repositories. Hence, the annotations must be updated on a regular basis. For those reasons, we require a database of all known splice variants and their exons. However, none of the published splice variant databases [25-46] permit explicit identification of microarray probes that distinguish splice variants. See Additional file 4 for a review of alternative splicing.

For that reason, we have developed (i) Evidence Viewer Database (EVDB), which provides a comprehensive, non-redundant collection of known human alternative splice forms, and (ii) SpliceMiner, a user-friendly tool for interactive and batch querying of EVDB. We constructed EVDB on the basis of data in the National Center for Biotechnology Information (NCBI) Entrez Gene [47] and NCBI Evidence Viewer (EV) [48]. EVDB maps gene symbols to a set of unique splice variants and identifies the exons present in each variant, along with transcript and genomic coordinates for each exon. SpliceMiner can be used to query EVDB by gene symbol, genomic coordinates, or probe sequence. Support for both interactive and batch queries is provided, and the SpliceMiner website provides high-throughput query functions that make it possible to integrate splice variant information into microarray analysis and design pipelines.

We will first describe EVDB in some detail and then present SpliceMiner. Further important information on the implementation of EVDB and SpliceMiner is included in Additional file 1 and Additional file 2, respectively.

## EVDB construction and contents

### Overview

EVDB is a relational database that describes all known splice variants of human genes for which GenBank [49,50] contains complete coding sequences. We constructed it on the basis of data in the NCBI Evidence Viewer (EV) and NCBI Gene but also used information from NCBI MapViewer [51], GenBank, RefSeq [52], Human Gene Nomenclature Committee (HGNC) gene symbols [53], and Enhanced Gene Ontology Database (EGOD) [54]. EVDB contains gene symbols, unique splice variants identified by accession ID, the exon composition of each variant, and both the genomic and transcript coordinates of each exon (Figure S1 in Additional file 3).

A goal of the project was to develop a splice variant database that conforms to a defined standard. NCBI Gene is a recognized standard for all gene-related data, is exhaustive with respect to known complete coding sequences, and is integrated with many other NCBI and non-NCBI data sources. EV, accessible through NCBI Gene, contains a number of different, useful types of information about a gene: the gene model, multiple sequence alignments, all RefSeq models, GenBank mRNAs, known or potential annotated transcripts, and ESTs [55]. We constructed EVDB primarily by converting the information in EV into a batch-queryable form. Currently, EVDB uses CDDSs to produce a non-redundant data set, but we are planning to include ESTs in a later release.

EVDB contains splice variant and exon coordinate data that are supported by complete transcript coding sequences. Genes that are predicted or based on EST evidence are represented in EVDB but without splice variant or exon coordinate data. The build of EVDB current at the time of publication of this paper (based on Human Genome Build 35.1) contains splice variant and exon composition data for 16,895 genes. As new builds of the human genome are released and additional complete coding evidence is produced, the number of genes in EVDB with splice variant and exon coordinate data will increase to match more closely the number of gene symbols in NCBI Gene.

### The EVDB build process

**Figure 1:**
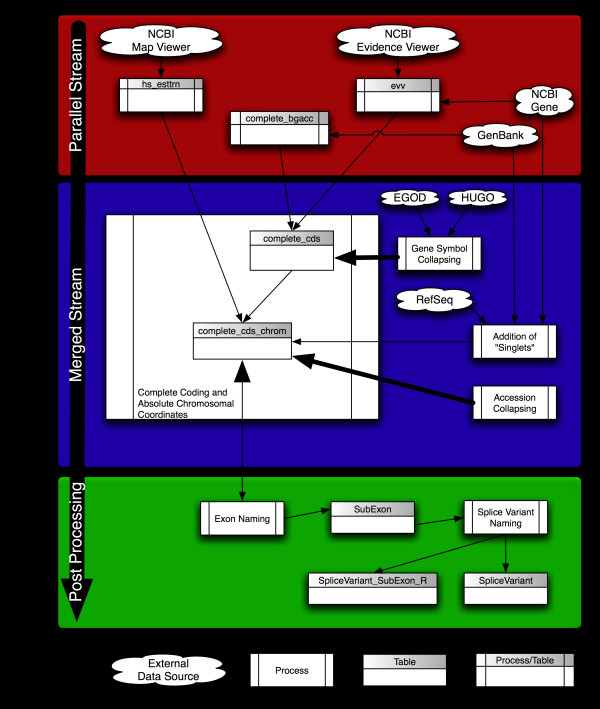
**EVDB construction process**. Files used for building EVDB are downloaded from "External Data" (see legend at bottom of figure). Downloaded data are converted into EVDB database tables by "Processes". Some tables are processes in themselves ("Process/Table");that is, a process with the same name was implemented to create the table. Small arrowheads on thin lines represent the flow of data. Large arrowheads represent processing within a specific table. Thick lines represent processes other than flow of data. Arrows meeting at one point represent database joins. Bi-directional arrows indicate that a method processes the data in the table and uses the data to create other tables. Algorithms for exon, sub-exon, and splice variant naming are beyond the scope of this paper and will be described elsewhere (Kahn *et al*., in preparation).

The EVDB build process is automated to facilitate updates as source data change. Figure 1 summarizes the process and provides a general schema for EVDB. The build process gathers data in parallel from EV, GenBank, and Map Viewer, using NCBI Gene as the source for an exhaustive list of gene identifiers (IDs; formerly "Locuslink" IDs). The downloaded GenBank files are then parsed to build a list of all accession IDs that represent complete coding sequences of human genes. Gene structure information, which includes identification of splice variants, is gathered from EV by a web robot. Finally, data from NCBI Map Viewer are used to determine chromosomal coordinates of each exon. Data from the parallel streams are then loaded into intermediate processing tables in the database.

After the parallel data-gathering streams have finished, a single merged-stream process creates two additional tables (*complete_cds *and *complete_cds_chrom*). To identify complete coding and RefSeq accession IDs, tables *evv *and *complcds_bgacc *are joined by accession ID to produce table *complete_cds*. Absolute chromosomal coordinates are assigned to each exon by joining tables *hs_esttrn *and *compete_cds *by contig, accession ID, and transcript coordinates to produce table *complete_cds_chrom*. Table *complete_cds_chrom *contains all of the data necessary to deduce splice variants.

In the current EVDB build, approximately 685 accession IDs map to multiple gene symbols. Those multiple mappings arise when there are alternative promoters for the same transcript. Since we are interested in the mapping of probes to splice variants, assignment of accession IDs to multiple gene symbols is a confounding factor. A gene-symbol collapsing algorithm removes that form of redundancy. Transcripts in multiple gene records are pooled into one non-redundant record when the algorithm finds at least one accession ID in common between genes. EGOD symbols were chosen over HGNC symbols because we plan to integrate EVDB with GoMiner [56,57] analysis, and GoMiner queries EGOD [54]; otherwise, HGNC symbols are chosen in preference to non-HGNC symbols.

Many gene symbols have more than one accession ID for a transcript. EVDB is intended to be a non-redundant database of splice variants, so repeated records with duplicate gene structure are filtered. An algorithm for filtering replicate accession IDs compares the chromosomal coordinates of each exon for all transcripts of a given gene symbol. Transcripts with identical exon coordinates are filtered. RefSeq accession IDs were chosen over redundant GenBank accession IDs.

Not all genes are represented in EV. We will refer to those genes as "MIAs." Many MIAs are not reviewed, are not validated, or are simply predicted but not experimentally verified. The EVDB build process includes a step that loads MIA symbols into EVDB. MIAs are included for completeness but lack gene structure information. As the data become available, MIAs will eventually be annotated and added to EV.

General algorithms and a more detailed description of the EVDB build process are provided in Additional file 1. Algorithms for naming exons, sub-exons, and splice variants (see Figure 1) are beyond the scope of the present work, but, in brief, the build process uses a novel naming convention that accommodates discovery of new splice forms and exon structures without the renaming of previously described exons (Kahn *et al*., in preparation). That naming convention, which identifies splice variants uniquely, is intended to facilitate integration of splicing information into other software tools and processes.

### Versioning and data asynchrony

Using the latest build of EVDB is not the best strategy for all research projects. Experimental results and software development may be based on a particular version of EVDB. Therefore, all entries in all tables are versioned for minor updates, and separate databases are implemented for each new build of the Human Genome. Methods for querying older versions of EVDB through the Web API will be provided.

### EVDB construction and quality control

EVDB was constructed using in-house Perl (Version 5.8.6) programs and PostgreSQL (Version 8.1). The Perl programs are modularly coded for each processing stream. Each subroutine in a module contains test subroutines and test data. Data and database integrity checks are also implemented.

### Contents of EVDB

The contents of EVDB are summarized in figures and tables in Additional file 3.

## SpliceMiner system architecture

SpliceMiner is a web interface/tool for querying EVDB. To facilitate deployment and support, we developed it on a platform consistent with existing NCI web-based systems. The system was constructed using open source tools that do not require license fees for production deployment. The technical details of the system architecture and implementation, and a schematic of the primary system components is displayed in Figure 1 of Additional file 2.

## Utility and discussion

### SpliceMiner

**Figure 2:**
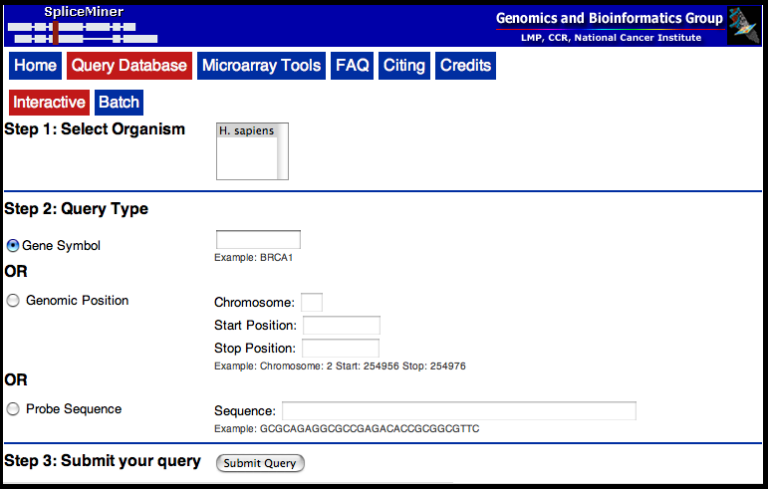
**Interactive SpliceMiner query**. The interactive query page allows the user to submit a SpliceMiner query by specifying a gene symbol, genomic coordinates, or a probe sequence.

**Figure 3:**
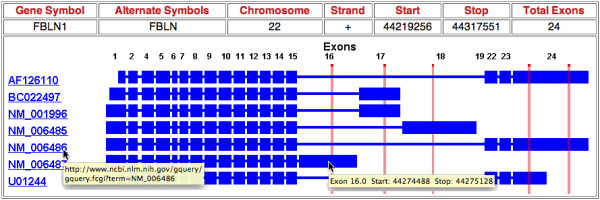
**Interactive SpliceMiner query results**. This figure is a composite of five separate interactive queries. Each query corresponds to a different Affymetrix HG-U133A Probe. The composite permits facile comparison of the exons that are targeted by each of the probes. For example, the probes for exons 16 and 18 uniquely identify the splice variants NM_006487 and NM_006485 respectively. In contrast, the probe for exon 17 identifies an unspecified mixture of splice variants BC022497 and NM_001996. Although that probe does not provide unique identification, it reduces the ambiguity from 7 splice variants to 2 splice variants. A somewhat more complex identification is afforded by the combined use of the two probes for exon 24. By difference, those probes in theory can uniquely identify the splice variant U01244. Variants are identified by accession ID, and a map of the exons in each variant is displayed. Exons are indicated by larger blue bars and are drawn to scale. Thin blue lines represent intron sequences but are *not *drawn to scale. If the query consists of a sequence or set of coordinates, a red vertical line identifies the matching location. A pop-up tool tip displays the URL query string that is invoked upon clicking on an accession ID. Another pop-up tool tip, which provides the exact genomic coordinates of the exon, is displayed by mousing-over the exon.

Both interactive and batch queries of EVDB are supported by SpliceMiner, a web tool and user-friendly (*e.g*. intuitive visualizations and hyperlinks to NCBI Entrez in interactive mode; user help *via *an FAQ section) graphical interface. The interactive portion of SpliceMiner (Figure 2) is intended for exploring splice variant information on particular genes or loci. A query is submitted as a gene symbol, genomic coordinates (*i.e*. chromosome, strand, start, end), or DNA sequence. The results of an interactive search are displayed graphically at the bottom of the query page (Figure 3). The results include information about the gene, its splice variants, and the exons that match the query symbol, location, or sequence. Gene symbol and chromosome position queries take less than one second; DNA sequence queries require searching a sequence database and take approximately 10 seconds.

Batch-request files can be pasted into the SpliceMiner text area or uploaded in text or zip file form. Small batch requests are processed immediately; larger batch requests are processed asynchronously, and the user is notified of completion *via *an email message containing a link to the results. Batch query results are presented in tabular form to support automated processing. A tab-delimited flat file is automatically generated and downloaded *via *a hyperlink in the email message (for large queries) or directly *via *a 'save-to-file' in the web browser (for small queries). Each line indicates the query string, gene, variant (identified by accession ID), exon, and both genomic and transcript coordinates. For gene queries, all variants and their exons are returned. For sequence or genomic coordinate queries, only the gene, variant, and exon combinations that are an exact match to the query are returned. For example, if the search sequence matches exon 4 of gene ACP1, only those variants containing exon 4 will be returned. A single large data file can be submitted to retrieve all splice variant data for a microarray in a single request, or a program can request splice variant data one probe or gene at a time.

### Use of SpliceMiner

The intent of SpliceMiner is to provide access to non-redundant splice variant and genomic data in EVDB, particularly for microarray research. Microarray design can be improved by augmenting probe placement decisions with knowledge of splice variant composition and exon structure. Similarly, analysis of microarray data from existing platforms can be improved by understanding the exon locations of the probes. The genomic positions of oligonucleotide probes may support inferences about the expression levels of specific splice variants. SpliceMiner queries can be integrated into microarray pipelines to add splice variant information.

The SpliceMiner web interface has been designed to facilitate integration with a variety of microarray pipelines. Pipelines that process large batch files as well as those that perform iterative gene by gene processing are supported. Integration with a batch processing pipeline is accomplished by submitting a single batch query file to SpliceMiner with a query line for each probe sequence or locus. Integration with an iterative process pipeline (*e.g*., microarray probe design) is accomplished by automating the query for a single sequence, symbol, or locus.

The sample Perl program (given in the FAQ section of the SpliceMiner web site at [58] or downloadable from [59]) illustrates one method for integrating SpliceMiner into a genomic pipeline. The LWP module is used to submit a web request to SpliceMiner for a gene symbol, genomic coordinate, or probe sequence query. The tab-delimited results are easily parsed with the Perl "split" function.

The "Probe Coverage" tool in SpliceMiner analyzes oligonucleotide microarray designs and provides a report of the splice variant/exon coverage. The report provides an overview of the transcript and exon coverage of each gene on the microarray. The first section shows how well the array does in covering each exon in a given gene with a probe; the second section presents additional information:

1. Whether variants have no probes;

2. Situations in which it is possible to evaluate probe-level signal to infer which variants are being expressed; and

3. Situations in which the probes in a probe set are likely to report differing signal values depending on the expression levels of different splice variants and the positions of the probes. If only a few probes related to one transcript are reporting signal, many analysis programs (*e.g*. MAS5) register an "Absent" score for the whole gene, and information about that gene's expression is lost.

Probe definitions are not used in the report because sequence data for the human genome continue to be refined, and probe sequences on older chips often no longer match their intended target gene. For that reason, sequence queries are performed by aligning [60] probes to a database of transcripts available in EVDB.

The Splice Variant/Exon Coverage Report demonstrates one of the benefits of applying SpliceMiner to microarray analysis. The report indicates the probes that can be used to estimate the expression of a specific splice variant. The report also flags potential problems that may lead to inaccurate gene-level expression values:

• inability to detect a splice variant

∘ none of the probes in the gene's probe set target any exons in that splice variant.

∘ *e.g*., occurs for 28% of the multi-variant genes represented on the Affymetrix HU_U95Av2 microarray.

• inconsistent detection of splice variants

∘ some probes in the gene's probe set target an exon that is missing in some of the splice variants (*e.g*., any of the probes in Figure 3).

∘ the downstream analysis algorithms (*e.g*., RMA or MAS5) assume that all of the probes in a gene's probe set target a consistent set of exons, but the input to the algorithms will violate this basic assumption.

∘ *e.g*., occurs for 42% of the multi-variant genes represented on the Affymetrix HU_U95Av2 microarray.

Reports for several common microarray platforms are provided on the website. They

1. give a summary, listed by gene symbol, describing those exons for which there is a probe and showing both the chromosomal and transcript coordinates where probes match each splice variant of the gene;

2. identify genes for which there is a probe that uniquely discriminates a splice variant; and

3. identify genes for which there is no probe for some or all splice variants.

A detailed description of the implementation of the web interface and related tools is provided in Additional file 2.

## Conclusion

SpliceMiner provides genomic researchers with access to EVDB, a source of non-redundant splice variant data that we designed for high-throughput analysis. Unlike NCBI's valuable Evidence Viewer, EVDB supports batch queries and queries across multiple genes. Because of its high-throughput capabilities, SpliceMiner is particularly useful for design and analysis of microarrays. SpliceMiner maps probes to splice variants, effectively delineating the variants identified by a probe. The addition of SpliceMiner to microarray pipelines provides a method for improving the accuracy of microarray results through inclusion of splice variant and exon composition data.

## Availability and requirements

The SpliceMiner website is available online at . SpliceMiner and EVDB data and results are made freely available to government, academic, and commercial users.

## Authors' contributions

ABK and MCR drafted the manuscript. ABK designed and implemented the EVDB database and EVDB build process. MCR designed and implemented the SpliceMiner tool and website and related tools described in this paper. HL, BRZ, DCJ, and JNW contributed to design of the EVDB database and website and revised the manuscript critically for important intellectual content. All authors gave final approval of the final version to be published.

## Supplementary Material

Additional File 4 Alternative splicing databases. This document provides a description of a large number of alternative splicing databases to provide context for the present study. Click here for file

Additional File 1 EVDB build process. This document provides a more detailed description of the process performed to construct EVDB. Click here for file

Additional File 2 SpliceMiner implementation. This document provides a system overview and software architecture description for SpliceMiner. Click here for file

Additional File 3 EVDB synopsis. This document provides an overview of the contents of EVDB. Click here for file
