# Supplementary material for: SpliceMiner: a high-throughput database implementation of the NCBI Evidence Viewer for microarray splice variant analysis
Source: BMC Bioinformatics. 2007 Mar 5;8:75. doi: 10.1186/1471-2105-8-75 (PMC1839109; doi:10.1186/1471-2105-8-75)
Supplement: Additional File 4 — Alternative splicing databases. This document provides a description of a large number of alternative splicing databases to provide context for the present study. [file 1471-2105-8-75-S4.doc]

Alternative Splicing Databases

There are at least 15 published splice variant databases, generated either by data mining data repositories (DMR) such as GenBank, Swiss-Prot, and Medline, or by comparison of sequence alignments (CSA). DMR alternative splicing databases include HS^3^D, ASDB, Xpro, the AEdb in ASD, and EVDB. CSA alternative splicing databases include ASTRA, ASAP, AltSplice and AltExtron in ASD, ASHESdb, EASED, ECgene, SpliceNest, ExInt, H-InvDB, MAASE, and FAST DB.

A description of each of the databases is given here to put the development of EVDB and SpliceMiner into context. We evaluate each database by two criteria:

- First, can it be used directly to map probe sequences to splice variants? That is, does it provide sequence alignment capabilities?
- If not, are the data within the database useful for mapping probe sequences to splice variants? That is, are variants delineated explicitly using high quality verified data (*i.e.* complete coding sequences).

This analysis illustrates the need for construction of EVDB. The description includes: contents, methods of splice variant delineation, high-throughput capabilities, and interface and querying functions. If querying features are available, an attempt is made to assess the database by submission of one gene (DDR1) and one Affymetrix probe from that gene (“probe:HG-U133A:1007_s_at:467:181”; CACCCAGCTGGTCCTGTGGATGGGA). DDR1 was selected by picking the first probe in the Affymetrix FASTA file *HG-U133A_probe_fasta* that corresponds to DDR1 as defined by Affymetrix and independent verification. As a well-characterized gene, DDR1 has records for 14 transcripts, including 3 RefSeqs and over 600 ESTs of varying quality. EVDB indicates that DDR1 contains 7 splice variants with respect to sub-exon structure of DDR1 transcripts. The EVDB also correctly maps the above Affymetrix probe to exon 20 on all 7 variants of DDR1. Although the description of each database includes all species covered by a database, only *Homo sapiens* (human) will be used as a comparator. This review was conducted in April, 2006. The Human Genome Build current at that time was 35.1.

The Alternative Splicing and Transcription Archives (**ASTRA**; <http://alterna.cbrc.jp/index.php> ) is a CSA database containing elementary patterns of alternative splicing and transcripts for six eukaryotes including humans [1]. The alternative splicing patterns were delineated by aligning full length cDNAs to the human genome and detecting variants by employing a novel algorithm that converts mappings between human genome sequences and cDNAs to binary descriptions [2]. A JAVA applet provides a visualization of the exon-intron structures of splice variants. Approximately 14,264 alternative splicing events of all types except alternative promoters are catalogued for humans. Submission of the gene DDR1 yielded 3 splice variants. No sequence querying or high-throughput capabilities were available.

The Alternative Splicing Annotation Project Database (**ASAP**; <http://bioinfo.mbi.ucla.edu/ASAP/> ) uses UniGene clusters of ESTs for realignment to the human genome [3]. The database can be queried by gene symbol, UniGene identifiers, gene title, or GenBank sequence identifier. Predefined queries are available for tissue and cancer types. The publication claims that more than 6,000 splice variants were detected. Submission of the gene DDR1 yielded 8 splice variants. No sequence querying capabilities were available. The database can be downloaded in whole for high-throughput analysis.

The EBI’s Alternative Splicing Database (**ASD**; <http://www.ebi.ac.uk/asd/> ) Project is composed of 3 databases: **AltSplice**, **AltExtron**, and **AEdb** [4]. Each database has a slightly different approach to cataloging alternative splicing events. AltExtron is a research and development pipeline, whereas AltSplice is a production pipeline. Both AltSplice and AltExtron realign ESTs and mRNAs to the genome and then identify splice variants by computational inspection of the alignments. Splicing events are classified as described in Section 1.2 (“Alternative splicing “) above. As of December 27, 2005 AltSplice consisted of 21,796 genes out of which 13,572 had at least one confirmed splicing event. Submission of gene DDR1 to AltSplice yielded 3 splice variants. AEdb is an ongoing collection of published experimental data on alternative splicing. Data collected includes nucleotide sequences, tissue specificity, developmental regulation, alternative exon function, and association with disease. In December, 2002 there were approximately 1100 entries. Submission of gene DDR1 to AEdb returned no results. No sequence querying capabilities were available for any of the databases in ASD. All three databases can be downloaded in whole for high-throughput analysis.

The Alternative Splicing Database (**ASDB**; <http://hazelton.lbl.gov/~teplitski/alt/> ) is composed of 2 divisions: proteins and nucleotides [5]. Approximately 1,922 protein sequences were collected from SWISS-PROT using full text search words “alternative splicing” and “varsplic”. Nucleotide sequences were collected from GenBank using the search term “alternative splicing”. Sequences were then clustered into common genes. Multiple sequence alignments were used to identify splice variants. Submission of gene DDR1 returned no results. No sequence querying or high-throughput capabilities were available.

The Alternatively Spliced Human Genes by Exon Skipping Database (**ASHESdb**; <http://sege.ntu.edu.sg/wester/ashes/> ) identifies 1,229 human genes that exhibit alternative splicing by exon skipping [6]. Variants are identified using realignments of full length cDNAs to the genome. The publication claims that the database integrates other data such as gene maps, gene structure, and tissue information. Submission of gene DDR1 returned no results. A BLAST query yielded inconclusive results for the DDR1 Affymetrix probe sequence; code names given splice variant results could not be resolved. However, it is unlikely that the correct result was returned as DDR1 was not found in the database when querying by gene, and BLAST results were not an exact match. No high-throughput capabilities were available.

The Extended Alternatively Spliced EST Database (**EASED**; <http://eased.bioinf.mdc-berlin.de/> ) is an online compendium of alternative splicing for several organisms including human [7]. Splice forms are defined by alignment of ESTs with mRNA using BLAST. Alignments that have more than one high-scoring pair are considered alternative spliced transcripts. Using over 3 million ESTs and 27,628 gene transcripts, EASED has identified 18,308 splice variants, suggesting that around 66% of genes have alternative splicing. The interface allows queries by GenBank accession, gene symbol, or phrases. Searches can be extended for the number of ESTs *per* splice site, developmental stages, cancer, or tissues in general. Search results in addition to splice variant structure include Ensembl gene data and a novel evidence graph that gives information about tissue specificity and developmental stages in which a variant is involved. Submission of the gene DDR1 yielded 10 splice variants. No sequence-querying or high-throughput capabilities were available.

The **ECgene** ( <http://genome.ewha.ac.kr/ECgene/> ) database uses the ECgene gene prediction algorithm to identify splice variants by EST clustering [8]. The database allows queries by UniGene, GenBank accession, and gene symbol. Many views of the resulting data are provided. including summary, gene structure, transcripts, functional annotation, SAGE by disease, and EST expression by tissue and disease. Using over 5 million ESTs and over 139,000 mRNAs, ECgene identified 9,312 spliced genes in humans. Submission of the gene DDR1 yielded 7 splice variants. No sequence querying capabilities were available. The database may be downloaded in whole for high-throughput analysis.

The Homo Sapiens Splice Sites Dataset (**HS^3^D**; <http://www.sci.unisannio.it/docenti/rampone/> ) is a downloadable database of Homo sapiens exon, intron and splice regions extracted from GenBank [9]. The author’s aim was to provide a standardized training set to assess the accuracy of computational approaches to gene identification and characterization. The authors have identified 4,523 exons and 3,802 introns in human nuclear DNA, including complete coding sequences (CCDS). Details about each exon or intron, including locus, start and end coordinates, sequence, and G+C content are reported. Windows of 140bp sequence around each splice site are also provided. No sequence-querying capabilities were available. The database can be downloaded for high-throughput analysis.

**SpliceNest** ( <http://splicenest.molgen.mpg.de/> ) uses mappings of EST consensus sequences to the human genome to identify splice variants [10]. Exon-intron boundaries are predicted from EST consensus sequences in GeneNest (<http://genenest.molgen.mpg.de/> ). Splice variants can be searched by chromosome or gene symbol. Submission of the gene DDR1 yielded hits on chromosomes 6 (13 consensus sequences) and 12 (1 consensus sequence). No sequence-querying or high-throughput capabilities were available.

The Exon/Intron Database (**ExInt**; <http://sege.ntu.edu.sg/wester/exint/index.html> ) stores information of all GenBank eukaryotic entries containing an annotated intron sequence [11]. Predicted gene structures are validated using EST data. A non-redundant set of the data is also provided. Statistics such as number of genes, exons and introns before and after purging, exon length distribution, intron length distribution, and intron phase distribution are available. For10,423 human genes ExInt identifies 76,383 exons and 65,960 introns. A keyword search as well as a BLAST search is available. Submission of the gene DDR1 did not yield any results. Submission of the Affymetrix probe also did not yield results. The database can be downloaded in whole for high-throughput analysis.

The **Xpro** (<http://origin.bic.nus.edu.sg/xpro/> ) database contains all eukaryotic protein-encoding DNA sequences contained in GenBank that have associated structural features listed in their records [12]. Xpro validates intron positions by re-alignment of a record’s sequence and EST sequences found in dbEST. Alternative splicing information is obtained during the validation process. A non-redundant set of the Xpro database is obtained by cross-referencing to the Swiss-Prot/TrEMBL and Pfam databases. The database currently contains information for 493,983 genes from different species. Of those genes, 351,918 are intron-containing, and 142,065 are intron-less. The publication states that Xpro is updated for each new GenBank release. A keyword search as well as a BLAST search is available. Submission of the gene DDR1 yielded 2 partial coding sequences. Submission of the Affymetrix probe yielded no results. The database can be downloaded in whole for high-throughput analysis.

The H-Invitational Database (**H-InvDB**; <http://www.jbirc.aist.go.jp/hinv/index.jsp> ) is a human gene database composed of 56,419 full-length cDNA clones representing 25,585 cDNA gene clusters [13]. For each cluster, the H-InvDB describes, among other representative data, the cluster’s gene structures, functions, and alternative splicing isoforms. Splice variants are delineated by pair-wise alignment of sequences. Submission of the gene DDR1 yielded 1 coding sequence with no variants. Submission of the Affymetrix probe yielded results consistent with the DDR1 CDS. The database can be downloaded in whole for high-throughput analysis.

The Manually Annotated Alternatively Spliced Events (**MAASE**; <http://maase.genomics.purdue.edu/> ) database system is comprised of two components: an alternative splicing event annotation tool, and a searchable database of annotated events that allows convenient export of information to aid in microarray design and data analysis. [14]. Splice variants are identified by aligning transcripts to the genome, then manually annotating the results. Out of 1,007 genes, MAASE displays information about 2,217 alternative splicing events. Submission of the gene DDR1 did not yield results. No sequence-querying or high-throughput capabilities were available.

The Friendly Alternative Splicing and Transcripts Database (**FAST DB**; <http://193.48.40.18/fastdb/> ) uses full and partial mRNA and EST sequences from Ensembl, UCSC, and GenBank for realignment to the genome [15]. FAST DB provides features such as an exon sequence query, probe alignment, and *in silico* PCR. Statistics on the database were unavailable, however the publication claims to use more than 80,000 transcripts to define more than 12,000 genes. FAST DB also has predefined queries for a list of 707 housekeeping genes defined by several other publications. Submission of the Affymetrix probe yielded correct results. Submission of gene DDR1 to FAST DB yielded a 3-exon gene with no splice variants. DDR1 is a 20-exon gene with at least 5 splice variants based on presence and absence in EVDB. No high-throughput capabilities were available.

**SpliceInfo** (<http://spliceinfo.mbc.nctu.edu.tw/> ) is a repository that collects data on alternative-splicing modes in the human genome [16]. The data are derived from comparisons of nucleotide and protein sequences available for a given gene. Additional features such as the tissue specificity, protein domains, GC ratio, and Gene Ontology are annotated computationally for each exon that is alternatively spliced. Out of 6,309 genes, SpliceInfo has identified 203,645 occurrences of alternative splicing events and cataloged each with respect to modes of alternative splicing. Querying is available by keywords including gene symbol. Submission of gene DDR1 to SpliceInfo yielded 1 hit with 7 splice variants. No sequence-querying or high-throughput capabilities were available.

Most of the alternative splicing databases focus on exploring the nature of splice variants. Some provide enough associations with additional related data sources (*e.g.* tissue specificity) to be useful for analysis of expression data in the context of alternative splicing. However, none of the databases specifically focus on de-convoluting the namespace of microarray probes with respect to splice variants. Methods could be added to most of the databases that would allow probes to be assigned to their proper targets. But many may be contaminated with low-quality EST sequences if filtering is not strict enough, or are not exhaustive with respect to all known complete coding sequence data of genes. The latter deficiency implies that variant data may be erroneous or that not all known transcript variants are available. Furthermore, the databases are redundant with respect to transcript data. Although some claim to provide non-redundant transcript datasets, they do not address the redundancy of transcripts in multiple genes (*e.g.,* alternative promoter genes). Most of the databases described are based on realignments of ESTs and/or mRNAs to the genome. Although that approach has identified many novel splice forms, the transcripts have not been experimentally verified. Data mining repositories (*e.g.,* EVDB), on the other hand, are based on defined standards in which the transcripts are more likely to have been verified. We obtained a wide range of results in our tests such as submission of the gene DDR1, suggesting that the approaches taken in formulation of the various databases are not equivalent. Using more than one source of data has the advantage of providing an independent verification but also introduces errors from multiple sources. Finally, although many of the databases allow downloads of data, none of them provide a high-throughput, queryable interface for determining probe identity.
