# Supplementary material for: SpliceMiner: a high-throughput database implementation of the NCBI Evidence Viewer for microarray splice variant analysis
Source: BMC Bioinformatics. 2007 Mar 5;8:75. doi: 10.1186/1471-2105-8-75 (PMC1839109; doi:10.1186/1471-2105-8-75)
Supplement: Additional File 1 — EVDB build process. This document provides a more detailed description of the process performed to construct EVDB. [file 1471-2105-8-75-S1.doc]

# EVDB Database Build Process

# Overview

The ultimate goal of the EVDB build process is to create a relational database that describes all known splice variants for humans. The splice variants are based on the gene structures of all known complete coding sequences from a defined standard. Future versions of this database will be expanded to include species other than human. EVDB contains the absolute chromosomal coordinates for all exons. The specific combination of exons for a complete coding transcript determines which splice variants for a gene are known. Aligning probes to gene transcripts determines the splice variants to which a probe hybridizes.

A goal of the project was to develop a splice variant database that conforms to a defined standard. Entrez Gene [1] is a recognized standard for all gene-related data, is exhaustive with respect to known complete coding sequences, and is integrated with many other NCBI and non-NCBI data sources. NCBI Evidence Viewer (EV) [2], accessible through NCBI Gene, contains the model of gene structure, multiple sequence alignments, and all evidence for a particular gene (Figure 1). EVDB is constructed primarily by converting the information in EV into a batch-queryable form. Entrez Gene and EV contain redundant and low quality EST data that are filtered during the EVDB build process.

The process of constructing EVDB is streamlined to facilitate updates. Because NCBI Gene is exhaustive with respect to complete coding sequences, so too is EVDB. NCBI Gene is a redundant database because there are multiple records (accessions) for a transcript. The redundant data are evidence for a gene and will therefore be displayed in the EV. The EVDB needs to be non-redundant with regard to transcript data so that splice variants can be defined. Therefore, the data from the EV are filtered at several stages.

There are three initial parallel processing streams, each of which creates a distinct table: *evv*, *complcds_bgacc*, *hs_esttrn*. The three streams merge into one processing stream to create two additional end-result tables: *complete_cds* and *complete_cds_chrom*. Many auxiliary tables are used for producing *complete_cds* and *complete_cds_chrom*. The auxiliary tables can be useful for other analyses but will be only briefly discussed here. Finally, post-processing of *complete_cds_chrom* names exons and identifies splice variants. An overview of the process is given in (Figure 2), which serves as a general schema for the EVDB.


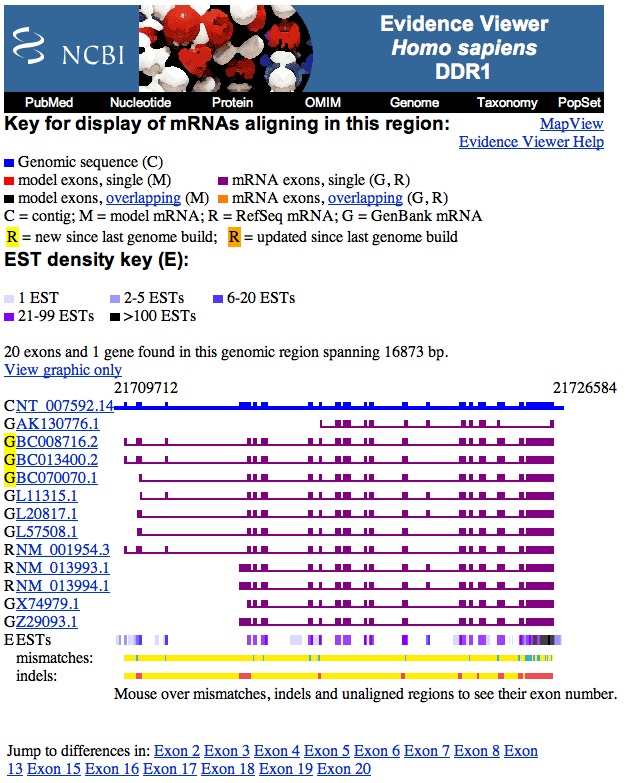


Figure 1 - An example of the NCBI Gene Evidence Viewer (for Gene DDR1).


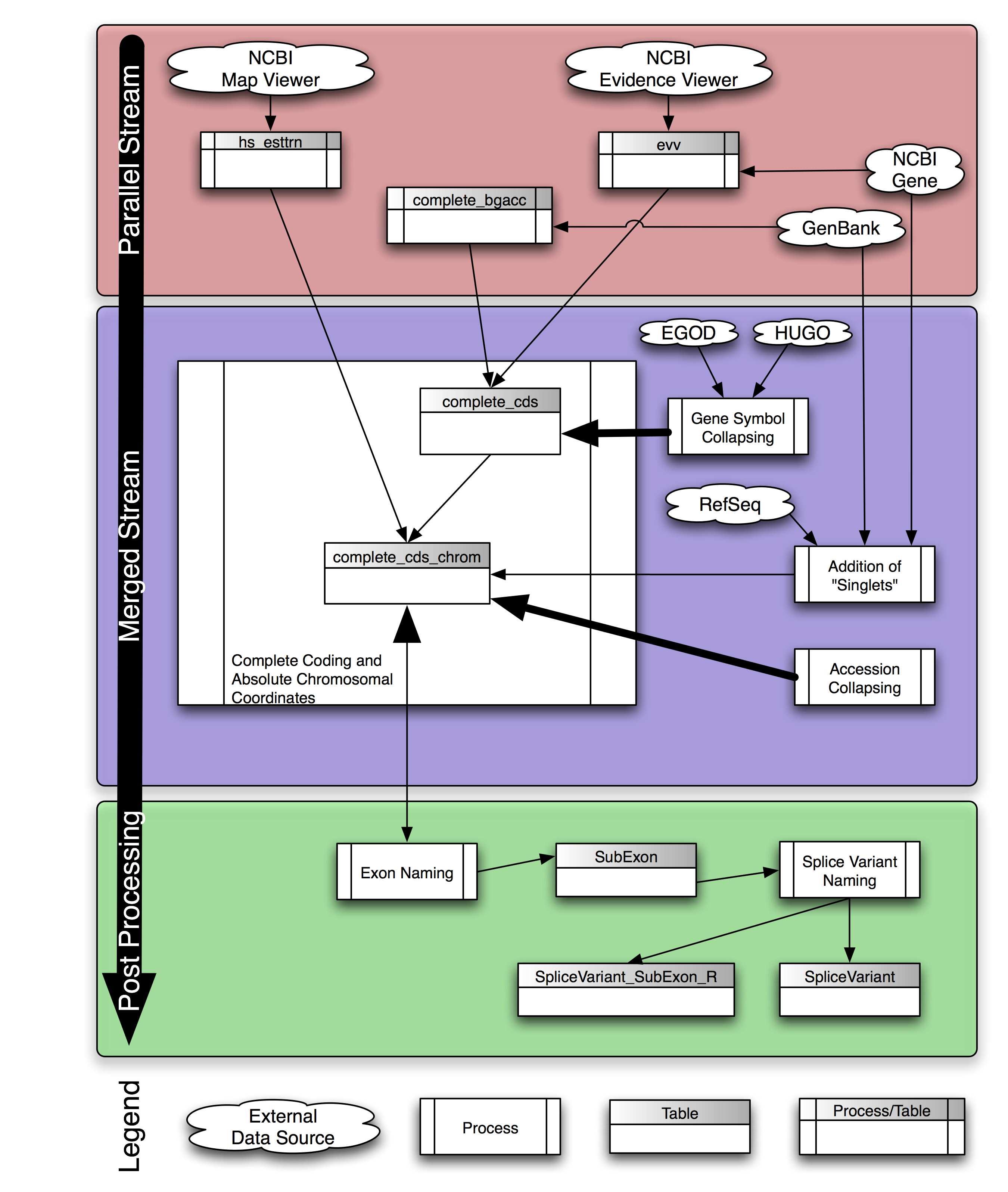


Figure 2 – The EVDB construction process.

An “External Data Source” is a set of files downloaded from the source. A “Process” is a set of methods that process the data. “Tables” are specific EVDB database tables. Some tables are also processes (“Process/Table”); that is, methods with the same name were implemented to create the tables. Small arrows represent the flow of data. Large arrows represent processing within a specific table. Arrows coming together at one point represent database joins. Arrows can be bi-directional indicating that a method both processes the data in the table and uses the data to create other tables. Although sub-exon, and splice variant naming processes and tables are displayed, they are beyond the scope of this paper, and will be described in a subsequent article. A general description of sub-exon and splice variant naming is given at the end of the section "Merged Stream: Exon Naming."

# Parallel Streams

## Stream evv

Unfortunately, the EV is not available in a downloadable table form and is only reachable through a complicated sequence of hyperlinks. Gene identifiers are obtained from the NCBI table “gene2accesssion”. A web-robot was constructed to query the EV given the list of all human gene identifiers. The general process is:

1. Download gene2accesssion from NCBI Gene FTP site ( <ftp://ftp.ncbi.nlm.nih.gov/gene/DATA/gene2accession.gz> );
2. Parse for Gene IDs (GID) of Human Genes;
3. For each GID, programmatically simulate querying NCBI Gene and save the resulting HTML source;
4. For each Gene HTML source, parse for NCBI EV URL;
5. Programmatically simulate clicking on EV hyperlink and save resulting HTML source;
6. Parse EV HTML source for the following variables: gene symbol, total exons, span of gene in base pairs, contig, (for each exon: accession, accession start and stop, and contig start and stop);
7. Save those variables in a text file and then pipe them into EVDB as the *evv* table.

## Stream complcds_bgacc

We need to determine which GenBank accessions are complete coding sequences so that we can exclude accessions from the *evv* table that are not complete coding sequences:

1. Download all primate GenBank files form the NCBI FTP site ([ftp://ftp.ncbi.nlm.nih.gov/genbank/gbpri[0-9]+.seq.gz](ftp://ftp.ncbi.nlm.nih.gov/genbank/gbpri%5B0-9%5D+.seq.gz); this URL is a regular expression for all gbpri seq files and is not intended to resolve correctly.);
2. Parse GenBank records and save all accessions that match the following constraints: “human” or “homo sapiens” and “complete cds” or “complete sequence”, but not “intronic transcript” or “BAC clone”.

Those accessions form the table *complcds_bgacc*.

## Stream hs_esttrn

We want to know the absolute chromosomal location of each exon in each gene. The NCBI Map Viewer table “hs_esttrn.md” contains that information but does not give the gene structure.

1. Download hs_esttrn.md from the NCBI FTP site
   (<ftp://ftp.ncbi.nih.gov/genomes/H_sapiens/maps/mapview/BUILD.35.1/hs_esttrn.md.gz> );
2. Import data directly into the database as table *hs_esttrn*.

As described next, we join *hs_esttrn* and a complete coding sequence version of the *evv* table (table *complete_cds*) in the Merged Stream.

# Merged Stream

### Complete Coding Sequences and Absolute Chromosomal Coordinates

A view of table *evv* that has only complete coding or RefSeq [3, 4] accessions is required. Since that view is queried extensively, it is a permanently indexed table (*complete_cds*). Table *complete_cds* is created by a join query on “accession” between table *evv* and table *complcds_bgacc*. Tables *evv* and *complete_cds* report exons as genomic contig coordinates. Since contigs can be overlapping, it is possible that exon coordinates for the same gene are reported on different contigs and therefore different coordinates for the same exon. Absolute chromosome coordinates are required to compare exon coordinates so that splice variants can be deduced. Table *hs_esttrn* has absolute chromosomal coordinates and contig coordinates but does not have gene structure data. Tables *complete_cds* and *hs_esttrn* are joined by “accession”, “contig”, and contig “start” and “stop”. A second join between *complete_cds* and *hs_esttrn* is conducted using the minus strand because genomic contig coordinates have reversed coordinates. The resulting table, *complete_cds_chrom*, has all the data required to deduce splice variants for a gene. In the sections below, this table will be filtered for redundant data, and exons will be named.

### Addition of MIAs

The EV is currently based on the data available at the time of the data freeze for the latest genome-build. Although the EV is updated often on a case-by-case basis, in general alignments are not generated after the release of a genome build. Therefore, a significant number of genes are missing from the EV. We refer to those genes as “MIAs”.

Because the database should be as complete as possible, MIAs are added to it. However, we do not consider the gene structure of MIA genes; EVDB is based on the EV, which we consider to be the standard for gene structure. Therefore, only the gene symbols, gene IDs, and a flag (“model”=0) to identify the record as having no additional data are added to *complete_cds_chrom*. As builds of the genome progress, the genes will eventually be annotated with RefSeqs and/or added to the EV. The process for adding MIAs is thus:

1. Download the NCBI Gene table “gene_info*”* (<ftp://ftp.ncbi.nih.gov/gene/DATA/gene_info.gz> )
2. Import the above data into a corresponding EVDB table (*gene_info*).
3. Create a table *MIA_GIDs* containing all MIA-gene IDs and gene symbols by joining *evv* and *gene_info* on “GID”.
4. For each record in *MIA_GIDs*, insert “GID”, “Symbol”, and “model”=0 into *complete_cds_chrom*.

## Gene Symbol Collapsing

In our preliminary analysis of EVDB, we have identified 685 accessions that are mapped to more than one gene symbol. That is, a number of gene symbols are “redundant” because they “contain” the same accession. Those data are stored in table *multiloci_acc*. The count of symbols per accession ranges from 2 to 22. Accessions are found in multiple genes because those accessions’ sequences have multiple alternative promoters, as described in [5]. Since one goal of this project is to map probes to splice variants, assigning accessions to multiple genes symbols confounds the analysis. Therefore, gene symbols that contain common transcripts are collapsed.

In general, the gene-symbol collapsing algorithm compares all accessions for all gene symbols. Genes are collapsed when the algorithm finds at least one accession in common between genes. GoMiner Enhanced Gene Ontology [6] symbols are chosen in preference to HGNC symbols because we plan on integrating the resulting data with GoMiner. HGNC [7, 8] symbols are chosen in preference to non-HGNC symbols.

Collapsing gene symbols is a procedural task and takes place in table *complete_cds*. Symbols are collapsed by setting “eliminate”=0. In addition to the table *multiloci_acc*, we also use 3 other auxiliary tables:

- *dist_genes_symb_proc* contains the accession-gene symbol relationship as well as the symbol membership count of an accession (Table 1);
- *gohugo* contains a list of HGNC gene symbols in the GoMiner enhancement [6] of the Gene Ontology (GO) [9, 10] database (EGOD);
- *hugo* contains the list of all HGNC [7, 8] gene symbols.

Table 1 Example of table *dist_genes_symb_proc* for gene ALF.

| **accession** | **symbol** | **symbol_member_cnt** | **proc** | **collapsed** |
| --- | --- | --- | --- | --- |
| NM_172311 | ALF | 1 | 1 | SALF |
| AF255310 | ALF | 2 | 1 | SBLF |
| AY026319 | ALF | 2 | 1 | SBLF |
| NM_006873 | ALF | 2 | 1 | SBLF |
| BC025991 | ALF | 2 | 1 | 0 |
| NM_006872 | ALF | 2 | 1 | 0 |
| NM_172196 | ALF | 1 | 1 | 0 |
| AF106857 | ALF | 2 | 1 | SALF |
| AF255310 | ALF | 2 | 1 | SALF |
| AY026319 | ALF | 2 | 1 | SALF |
| BC025991 | ALF | 2 | 1 | SALF |
| NM_006872 | ALF | 2 | 1 | SALF |
| NM_006873 | ALF | 2 | 1 | SALF |
| AF026169 | ALF | 1 | 1 | SALF |
| AF106857 | ALF | 2 | 1 | 0 |

The symbol-collapsing algorithm proceeds as illustrated in the following pseudo-code:

Update tables *gohugo* and *hugo* for the latest builds;

Construct table *dist_genes_symb_proc* by joining *complete_cds*
 and *multiloci_acc*;

Order *dist_genes_symb_proc* by gene symbol then by accession;

For each distinct accession-symbol combination in *dist_genes_symb_proc*
 with symbol member count>1 and processed=FALSE

{

Get all gene symbols that contain this accession;

If any of these gene symbols is in the set of EGOD symbols

Arbitrarily choose any one EGOD symbol as the representative symbol;

Else if any of these gene symbols is in the set of HGNCs

Arbitrarily choose any one HGNC symbol as the representative symbol;

Else

Arbitrarily choose any one symbol as the representative symbol;

Endif

Update *dist_genes_symb_proc* for all symbols in this symbol set

Set collapsed = the symbol being collapsed;

Set symbol = the representative symbol;

Set processed = TRUE;

}

For each record in *dist_genes_symb_proc* with symbol member count>1
 and processed=TRUE

{

Get accession (*a*), symbol (*s*), and collapsed (*coll*);

Update *complete_cds*

Set eliminate=TRUE where accession=*a* and symbol!=*s*;

If collapsed!=0

Set collapsed=*coll* and symbol=*s* where accession=*a*
 and symbol=*coll*;

}

### Accession Collapsing

One of the goals of this project is to create a non-redundant database of gene transcripts so that splice variants can easily be deduced. However many gene symbols have more than one record for a transcript in GenBank. Since another goal of the project is to map probes to splice variants, assigning probes to multiple transcripts of the same structure confounds the analysis. Therefore, transcripts are collapsed based on transcript structure so that proper comparisons can be made.

In general, the accession-collapsing algorithm compares all exon chromosomal coordinates of all transcript accessions for each gene symbol. A transcript is defined as unique if its set of exon coordinates is unique. Transcripts that have identical exon coordinates are collapsed. RefSeq accessions are chosen as representative accessions in preference to GenBank accessions.

Table *complete_cds_chrom* contains all gene structure data for all accessions. The table contains gene “symbol”, “accession”, “exon” number, chromosome “start”, chromosome “end”, “processed”, “eliminate”, and eliminate “isoform”. Ordering the table by “symbol”, “exon”, “start”, “end”, and “accession” allows the systematic processing of transcript isoforms (Table 2).

Table 2A subset of table *complete_cds_chrom* for gene symbol ‘ALF’, fully processed for both symbol and accession collapsing.

| **symbol** | **exon** | **accession** | **start** | **end** | **eliminate** | **collapsed** | **isoform** | **isoforms** | **processed** |
| --- | --- | --- | --- | --- | --- | --- | --- | --- | --- |
| ALF | 1 | AF026169 | 48707810 | 48707876 | 0 | SALF | 1 | [AF026169] | 1 |
| ALF | 1 | NM_172311 | 48707810 | 48707876 | 0 | SALF | 0 | [AF026169] | 1 |
| ALF | 1 | AF255310 | 48719414 | 48721353 | 1 | SBLF | 0 |  | 1 |
| ALF | 1 | NM_006873 | 48719414 | 48721353 | 1 | SBLF | 0 |  | 1 |
| ALF | 1 | AY026319 | 48719424 | 48721353 | 1 | SBLF | 0 |  | 1 |
| ALF | 1 | NM_006872 | 48756599 | 48756685 | 0 | 0 | 0 |  | 1 |
| ALF | 1 | NM_172196 | 48756599 | 48756685 | 0 | 0 | 0 |  | 1 |
| ALF | 1 | BC025991 | 48756628 | 48756685 | 0 | 0 | 0 |  | 1 |
| ALF | 1 | AF106857 | 48756650 | 48756685 | 0 | 0 | 0 |  | 1 |
| ALF | 2 | AF026169 | 48719377 | 48721353 | 0 | SALF | 1 | [AF026169] | 1 |
| ALF | 2 | NM_172311 | 48719377 | 48721353 | 0 | SALF | 0 | [AF026169] | 1 |
| ALF | 2 | AF255310 | 48719414 | 48721353 | 0 | SALF | 0 |  | 1 |
| ALF | 2 | NM_006873 | 48719414 | 48721353 | 0 | SALF | 0 |  | 1 |
| ALF | 2 | AY026319 | 48719424 | 48721353 | 0 | SALF | 0 |  | 1 |
| ALF | 2 | AF255310 | 48730443 | 48730645 | 1 | SBLF | 0 |  | 1 |
| ALF | 2 | AY026319 | 48730443 | 48730645 | 1 | SBLF | 0 |  | 1 |
| ALF | 2 | NM_006873 | 48730443 | 48730645 | 1 | SBLF | 0 |  | 1 |
| ALF | 2 | AF106857 | 48759641 | 48759742 | 0 | 0 | 0 |  | 1 |
| ALF | 2 | BC025991 | 48759641 | 48759742 | 0 | 0 | 0 |  | 1 |
| ALF | 2 | NM_006872 | 48759641 | 48759742 | 0 | 0 | 0 |  | 1 |
| ALF | 2 | NM_172196 | 48759641 | 48759742 | 0 | 0 | 0 |  | 1 |

We rely on 2 auxiliary tables to facilitate the collapsing process:

- *ud_symb_gene_xnum* contains all exons concatenated as a string, “exon_nums” (essentially splice variants deduced by using presence or absence of an exon) for each “accession” for each gene “symbol”;
- *distinct_exon_coords* contains all distinct exons coordinates, “start” and “stop”, for each “exon” for each “symbol”;

Redundant accession isoforms are eliminated by setting “isoform”=1 in *complete_cds_chrom*.

The accession-collapsing algorithm proceeds as illustrated in the following pseudo-code:

While there are still accessions un-processed {

Get the next accession (*a*), gene symbol (*s*), exon number (*e*), and
 chromosome start (*t*), and end (*n*) from *complete_cds_chrom* where processed=0 and eliminate=0;

If there is only 1 accession in *complete_cds_chrom* with *t* and *n*

Update *complete_cds_chrom*

Set processed=1 where accession=*a;*

Get the set of all exons, *E*, for *a* in *s* from *ud_symb_gene_xnum*;

Get the set of all accessions, *A*, that have *E* in *s*;

If the length of *A*=1

Update *complete_cds_chrom*

Set processed=1 where accession=*a;*

For *i* in *E* do {

Get the number of accessions (*u*), that have exon *E_i_*
 from *distinct_exon_coords*;

If *u*=0, ERROR

If *u*=1

If *E_i_* occurs in all accessions of *A*, next;

If *E_i_* occurs in only *a*

Update *complete_cds_chrom*

Set processed=1 where accession=*a*;

Else

Get the chromosomal start, *E_t_* , and end, *E_u_* , for *E_i_*
 from *distinct_exon_coords*;

If there is only 1 accession in *complete_cds_chrom*

with *E_t_* and *E_u_*

Update *complete_cds_chrom*

Set processed=1 where accession=*a*;

Endif

If *i*=length(*E*)

If any of the accessions in *A* are RefSeqs

Arbitrarily choose any RefSeq as the representative

accession, *r*;

Else

Arbitrarily choose any accession as the representative

accession, *r*;

Endif

Remove *r* from *A*;

Update *complete_cds_chrom*

Set processed=1, isoform=1, isoforms=*E*

where accession in *A*;

Set processed=1, isoform=0, isoforms=*E*

where accession= *r;*

Endif

}

}

### Exon Naming

A splice variant is comprised of a set of exons with at least one exon that has unique chromosomal coordinates. A gene’s overall structure, the *constitutive* [5] *gene structure*, is the composite of all overlapping exons from all splice variants. A variant’s exon may differ significantly from its corresponding constitutive exon. *Constitutive exons*, the composite of all overlapping exons, may contain internal acceptor and/or donor sites that give rise to variants. Any of the resulting constitutive sub-exons may be functional. With the above in mind, it is advantageous to name both variant exons and variant sub-exons.

Naming of variant exons is straightforward. For each gene symbol, sort the exon chromosomal start and end coordinates; for each set of sorted exon coordinates, append to the exon number the serially increasing index of the number of exon isoforms. For example, if exon 2 has 3 isoforms, the variant exons would be named 2.0, 2.1, and 2.2.

The exon-naming algorithm proceeds as illustrated in the following pseudo-code:

For each gene symbol {

Sort all exons by exon chromosomal start and end;

Set exon_isoforms=0;

For each set of identical exon numbers {

Get the set, *C*, of distinct chromosomal coordinates,
 start (*s*) and end (*e*);

Set exon_name=exon number.exon_isoforms;

Update *complete_cds_chrom*

Set exname= exon_name;

exon_isoforms++;

}

}

Algorithms for sub-exon and splice variant naming (also displayed in Figure 2) are beyond the scope of this paper, and will be described in a subsequent article. The sub-exon naming process provides a unique naming scheme that accommodates the discovery of new splice forms and exon structures without renaming of previously described exons. The splice variant naming process identifies splice variants uniquely and is intended to facilitate integration of the information into other software tools and processes.

# References
