# Supplementary material for: SpliceMiner: a high-throughput database implementation of the NCBI Evidence Viewer for microarray splice variant analysis
Source: BMC Bioinformatics. 2007 Mar 5;8:75. doi: 10.1186/1471-2105-8-75 (PMC1839109; doi:10.1186/1471-2105-8-75)
Supplement: Additional File 2 — SpliceMiner implementation. This document provides a system overview and software architecture description for SpliceMiner. [file 1471-2105-8-75-S2.doc]

# SpliceMiner Implementation

# System Architecture

SpliceMiner is a web interface/tool for querying EVDB. To facilitate deployment and support, we developed it on a platform consistent with existing NCI web-based systems. The system was constructed using open source tools that do not require license fees for production deployment. A schematic of the primary system components is displayed in Figure 1.

Browser

HTML

HTML

Remote Users

Internet

NCI

Solaris Server

EV

Database

Tomcat

mySQL

SQL

Program

E-Mail

Client

User

Disk

Report

BLAT

Query

Results

Custom BLAT DB

SMTP

Server

Batch

Process

Launch

Batch Completion E-Mail

Batch Completion E-Mail

Temp Results Store

Report Zip File

Figure 1: System Architecture

System components include:

- **Browser** – Standard web browsers are used to access the system *via* the internet. The tools should be functional in any browser but some “tooltip” pop-ups on gene map exons may not work on non-IE browsers.
- **Program** – Automated pipeline programs can access the system’s splice variant information using the html request/response protocol.
- **E-Mail client** – For batch jobs that are executed asynchronously, e-mail notifications are sent to users when jobs are complete.
- **User Disk** – Large batch reports are downloaded as zip files to user disk storage.
- **Solaris Server** – Solaris UNIX servers are used at NCI for web-based tools. SpliceMiner is implemented in Java and runs under Tomcat so it can be deployed on any Windows or UNIX system that supports Java.
- **Tomcat** – Apache Tomcat V5.0 is used as the web server for the application. It is an open source product and provides support for J2EE standard Java Servlets and JavaServer Pages.
- **mySQL** – MySQL is an open source relational database. EVDB tables are stored in the MySQL version 4.1.14 database server.
- **BLAT** – BLAT is used by EVDB web tools to match query sequences to genes and transcript locations. BLAT is run against a local custom BLAT database containing all the sequences for transcripts in EVDB.
- **Batch Process** – When users submit large batch query requests, a Java process is launched to run the batch request as a background process.
- **SMTP Server** – Batch processes notify users *via* email messages that their jobs are done. Any SMPT email server can be used. Authentication with a configurable user/password is provided with the outbound email.

## Software Design

SpliceMiner was constructed in an object-oriented (OO) fashion to create reusable common components. OO design was also used to create abstractions that cleanly divide major components so that individual components can be modified without requiring changes to the rest of the system. The iterative nature of the development effort required many significant changes to the system, and the OO design provided the appropriate flexibility to support those changes.

A high level diagram of the major software components of the system is presented in Figure 2. The flow starts with a user request received as an HTML Request object. Requests are handled by servlets that verify and forward the request to the appropriate Java Server Page (JSP). The JSP calls Utility Object functions as needed to satisfy the request. Utility objects work with BLAT and SpliceMiner to perform a search and to create Data Objects that contain the results. The Data Objects are returned to the JSP, which performs the processing necessary to format and present the results as an HTML page. For large batch requests, a batch process is launched by the servlet (rather than being forwarding as a request to a JSP). The batch process uses the utility objects to perform the requested user search and then creates a zip file of results placed in a temporary storage area on the webserver. The batch process then uses another utility object to email a completion notice with a link to the results file.

HTML Request

NCI

Presentation Layer

JSP

SQL

Servlets

Data Objects

Query

Result

Gene

Utility Objects

EVV_DB

HTML Reply

Variant

Exon

BLAT

Result

Search

Result

BLAT

Search

Config

BLAT

Properties

EVV

ImageGen

EVV

EMail

Batch Processes

Position

Batch

E-Mail

1

2

3

4

Figure 2: Software Objects and High Level Interactions

The following is a brief description of the software objects in the system:

- **Servlets** – There are currently three servlets: Search.java handles all interactive queries, Batch.java handles all batch query requests, and MicroArray.java handles the microarray-specific requests. Servlets generally verify user input and call the appropriate JSP. Sometimes they may call utility functions to perform processing (*e.g.* a BLAT search) prior to forwarding the request to the JSP. Servlets also handle file uploads, zip decompression, and text areas for batch requests. The org.apache.commons.fileupload was very useful for implementing file upload processing, and the built-in java.util.zip supports zip file processing. The process was implemented such that text areas, text files, and zip files are all opened as an InputStream and passed to utility functions as a BufferedReader so that the same utility function can be read directly from a text area string, plain text file, or zip file. Finally, batch servlets decide if batch requests are too large for interactive processing and, if so, launch an asynchronous batch process. That decision is made by a configurable batch size stored in the EVV.properties file.
- **Utility Objects** - The purpose of the utility objects is to encapsulate the interaction with external components and provide simple, reusable objects for common functions.
  - **EVV_DB** - performs all relational database queries using jdbc and SQL. The details of the database schema and SQL calls are handled by this object. Data Objects containing results are returned to the caller.
  - **EVV_Batch_DB** - is another DB utility object that is optimized for high volume DB interaction. It holds onto DB connections to minimize connection overhead, returns results in a compact format, and performs fast bulk loads of temporary results tables.
  - **BLAT Search** -This object contains the logic to prepare a BLAT query file, launch a stand-alone BLAT process, wait for results, and parse results once the BLAT search is complete. A vector of search result data objects is returned by the BLAT Search object.
  - **Config** - This singleton provides configuration information to any other component of the system needing user-configurable run time parameters. Currently its backing store is a Java properties file that must be in the classpath at runtime.
  - **EVV_ImageGen** – This utility function is able to take a gene data object and draw an image of the variants and exons of the gene suitable for display in the browser. Java AWT and Graphics2D functions are used to draw the image. Exon x,y positions are returned in the gene data object so that the JSP can add pop up “tooltip” information on the image.
  - **EVV-EMail** - This utility function sends email messages. Message and target address information are passed in. It is able to use any SMTP server to send the email and can perform authentication if required. SMTP configuration information is in the EVV.properties file.
  - **Log4J** – This open source package is used by all system components to log error information and debug messages to a central log file.
- **Data Objects** - These are simple data holder objects that are often returned by utility objects in a vector. In addition to holding data, they may contain simple helper functions (*e.g.* routine to build a consensus exon map for a gene or find the overall start/stop position for a gene). Gene data objects hold a collection of variant data objects, which in turn hold a collection of exons. This data structure is used by the JSP to draw gene map images or display summary results. Batch queries often use a more compact data object, omitting nested object structures to improve performance and use less memory.
- **JSPs** – Java Server Pages are used as the presentation layer of the application. They call utility functions, retrieve collections of data objects, and build HTML pages for presenting results. Gene.jsp is the interactive query page and displays interactive query results. Batch.jsp is the batch query page that handles batch query submission. Batch results are returned as either a simple text page or zip file with text results. The results are formatted as simple text files to facilitate automated result processing. MicroArrayPos.jsp submits microarray probe sequence data files. Basic JSP pages were created for the site home page, citations page, and credits page. The look and feel of the website was designed to be consistent with other NCI Genomics & Bioinformatics Group tools such as GoMiner. A common stylesheet is used to achieve that objective.
- **Batch Processes** – When the Batch servlet determines that a batch request has exceeded the size threshold for immediate processing, a custom stand-alone Java process is launched to process the request asynchronously. The Java class MicroArrayPositionBatch is the batch process. It can handle several types of batch queries. It runs BLAT or EVDB searches, formats the results into zipped report files, and emails the user when the job is complete. Report files are placed in temporary storage on the web server, and the user is given a link to download the report.
- **Data Load Utilities** – Although not part of the EV Web Interface *per se*, a few data load programs are required for data updates. EVDB is rebuilt as updates to the related genome database are released. Data Load scripts are required to load the data into MySQL and to build the appropriate indices to support the heavily used query paths. Additionally, the custom BLAT database needs to be rebuilt each time EVDB is updated. The sequence records for each transcript in EVDB must be extracted from RefSeq and GenBank datasets and integrated into a FASTA file that serves as the EV custom BLAT DB. Perl and SQL scripts automate that process and verify the contents of the BLAT DB.
