# Supplementary material for: SpliceMiner: a high-throughput database implementation of the NCBI Evidence Viewer for microarray splice variant analysis
Source: BMC Bioinformatics. 2007 Mar 5;8:75. doi: 10.1186/1471-2105-8-75 (PMC1839109; doi:10.1186/1471-2105-8-75)
Supplement: Additional File 3 — EVDB synopsis. This document provides an overview of the contents of EVDB. [file 1471-2105-8-75-S3.doc]

Synopsis of EVDB

+--------+-----------+-------+----------+----------+------+--------+--------+--------+

| **symbol** | **acc**  | **exon_** | **accexon_** | **accexon_** | **chr** | **strand** | **chr_** | **chr_** |

| | | **num** | **start** | **stop** | | | **start** | **stop** |

+--------+-----------+-------+----------+----------+------+--------+--------+--------+

| ACP1 | NM_177554 | 1 | 1 | 112 | 2 | + | 254896 | 255007 |

| ACP1 | NM_177554 | 2 | 113 | 186 | 2 | + | 261866 | 261939 |

| ACP1 | NM_177554 | 3 | 187 | 215 | 2 | + | 262037 | 262065 |

| ACP1 | NM_177554 | 4 | 216 | 329 | 2 | + | 262192 | 262305 |

| ACP1 | NM_177554 | 5 | 330 | 391 | 2 | + | 265140 | 265201 |

| ACP1 | NM_177554 | 6 | 392 | 497 | 2 | + | 266980 | 267085 |

| ACP1 | NM_177554 | 7 | 498 | 1554 | 2 | + | 267227 | 268283 |

| ACP1 | NM_007099 | 1 | 1 | 112 | 2 | + | 254896 | 255007 |

| ACP1 | NM_007099 | 2 | 113 | 186 | 2 | + | 261866 | 261939 |

| ACP1 | NM_007099 | 4 | 187 | 300 | 2 | + | 262192 | 262305 |

| ACP1 | NM_007099 | 5 | 301 | 362 | 2 | + | 265140 | 265201 |

| ACP1 | NM_007099 | 6 | 363 | 468 | 2 | + | 266980 | 267085 |

| ACP1 | NM_007099 | 7 | 469 | 1525 | 2 | + | 267227 | 268283 |

| ACP1 | NM_004300 | 1 | 1 | 112 | 2 | + | 254896 | 255007 |

| ACP1 | NM_004300 | 2 | 113 | 186 | 2 | + | 261866 | 261939 |

| ACP1 | NM_004300 | 3 | 187 | 300 | 2 | + | 262037 | 262150 |

| ACP1 | NM_004300 | 5 | 301 | 362 | 2 | + | 265140 | 265201 |

| ACP1 | NM_004300 | 6 | 363 | 468 | 2 | + | 266980 | 267085 |

| ACP1 | NM_004300 | 7 | 469 | 1525 | 2 | + | 267227 | 268283 |

+--------+-----------+-------+----------+----------+------+--------+--------+--------+

Figure S1 - Sample EVDB data

EVDB contains information on non-redundant splice variants identified by accession ID for each gene symbol. Each variant contains one entry *per* exon. The exon entries identify the transcript coordinates and genomic coordinates of the exon. “symbol” is the HGNC symbol, “acc” is the GenBank accession ID, “exon_num” is the exon number as listed in EV, “accexon_start” and “accexon_stop” are the starting and ending positions, respectively, of the exon in transcript coordinates, “chr” is the chromosome ID, “strand” is the chromosomal strand, and “chr_start” and “chr_stop” are the starting and ending positions, respectively, of the exon in chromosomal coordinates.

Table S1 - Counts of various entities contained in EVDB

| Gene Symbols | 32,789 |
| --- | --- |
| Genes with Models | 17,161 |
| Genes without Models (*i.e.,* MIAs) | 15,894 |
| Genes after Collapsing Symbols | 16,958 |
| Accession IDs with Complete Coding Sequences | 60,404 |
| Accession IDs after Collapsing Transcripts | 59,454 |
| Genes with Splice Variants in EVDB | 6,552 |

6,552 (39%) genes that contained splice variants were identified based on the pattern of presence and absence of exons. Most genes had between one and three splice forms (Figure S2). “Genes with Splice Variants in EVDB” means genes with two or more non-redundant GenBank records that are designated as “complete coding sequences.”


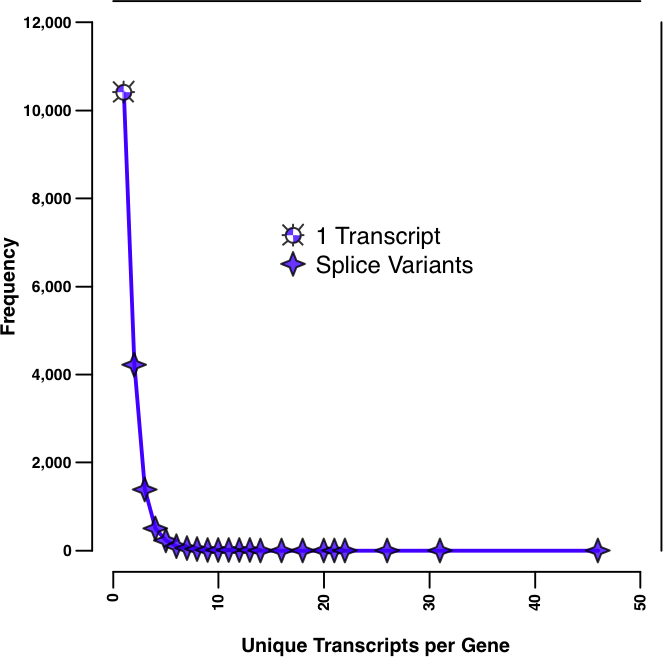


Figure S2 - Frequency distribution of unique transcripts per gene

Genes with one transcript comprise 62% of all transcripts; genes with splice variants (*i.e.* genes with two or more non-redundant GenBank records that are designated as “complete coding sequences”) comprise the remaining 38%. The data are presented numerically in Table S2 to facilitate interpretation of the transcripts present at low frequency.

Table S2 - Frequency Distribution of Unique Transcripts *per* Gene

| **Unique Transcripts**  ***per* Gene** | **Frequency** | **Normalized Frequency** |
| --- | --- | --- |
| 1 | 10406 | 6.14E-01 |
| 2 | 4219 | 2.49E-01 |
| 3 | 1378 | 8.13E-02 |
| 4 | 504 | 2.97E-02 |
| 5 | 226 | 1.33E-02 |
| 6 | 92 | 5.43E-03 |
| 7 | 53 | 3.13E-03 |
| 8 | 28 | 1.65E-03 |
| 9 | 13 | 7.67E-04 |
| 10 | 10 | 5.90E-04 |
| 11 | 5 | 2.95E-04 |
| 12 | 7 | 4.13E-04 |
| 13 | 5 | 2.95E-04 |
| 14 | 3 | 1.77E-04 |
| 16 | 2 | 1.18E-04 |
| 18 | 1 | 5.90E-05 |
| 20 | 1 | 5.90E-05 |
| 21 | 1 | 5.90E-05 |
| 22 | 1 | 5.90E-05 |
| 26 | 1 | 5.90E-05 |
| 31 | 1 | 5.90E-05 |
| 46 | 1 | 5.90E-05 |
| Total | 16,958 | 1.00 |
